# 17-β-Estradiol induces spreading depression and pain behavior in alert female rats

**DOI:** 10.18632/oncotarget.23141

**Published:** 2017-12-09

**Authors:** Alexander J. Sandweiss, Karissa E. Cottier, Mary I. McIntosh, Gregory Dussor, Thomas P. Davis, Todd W. Vanderah, Tally M. Largent-Milnes

**Affiliations:** ^1^ Department of Pharmacology, College of Medicine, University of Arizona, Tucson, Arizona 85724, USA; ^2^ School of Behavioral and Brain Sciences, University of Texas at Dallas, Richardson, Texas 75080, USA

**Keywords:** migraine, trigeminal, headache, neuroendocrine, aura

## Abstract

**Aims:**

Test the putative contribution of 17-β-estradiol in the development of spreading depression (SD) events and head pain in awake, non-restrained rats.

**Main Methods:**

Female, Sprague-Dawley rats were intact or underwent ovariectomy followed one week later by surgery to place electrodes onto the dura to detect epidural electroencephalographic activity (dEEG). dEEG activity was recorded two days later for 12 hours after systemic administration of 17-β-estradiol (180 μg/kg, i.p.). A separate set of rats were observed for changes in exploratory, ambulatory, fine, and rearing behaviors; periorbital allodynia was also assessed.

**Key Findings:**

A bolus of 17-β-estradiol significantly elevated serum estrogen levels, increased SD episodes over a 12-hour recording period and decreased rearing behaviors in ovariectomized rats. Pre-administration of ICI 182,780, an estrogen receptor antagonist, blocked 17-β-estradiol-evoked SD events and pain behaviors; similar results were observed when the antimigraine therapeutic sumatriptan was used.

**Significance:**

These data indicate that an estrogen receptor-mediated mechanism contributes to SD events in ovariectomized rats and pain behaviors in both ovariectomized -and intact- rats. This suggests that estrogen plays a different role in each phenomenon of migraine where intense fluctuations in concentration may influence SD susceptibility. This is the first study to relate estrogen peaks to SD development and pain behaviors in awake, freely moving female rats, establishing a framework for future preclinical migraine studies.

## INTRODUCTION

Migraine is one of the most common neurological disorders in the world, affecting 14.2% of US adults [1]. Overall, migraineurs spend nearly 2.5 times more than non-migraineurs in direct healthcare costs, and recurrent episodes result in approximately 11 days of missed work that costs up to $10,000/case annually [2, 3]. Roughly two-thirds of migraineurs are female suggesting a potential role for estrogens in migraine pathophysiology [4], yet very few preclinical studies have used female subjects [5, 6].

According to the International Classification of Headache Disorders, two types of migraine exist: migraine with and without aura. Migraine with aura (MA), a focal neurological disturbance exhibiting predominantly visual symptoms, affects one-third of all migraineurs [7–9]; this phenomenon is closely associated with spreading depressions (SD). SD events are self-propagating waves of membrane depolarization that travel at rates of 2-5 mm/min followed by a negative shift of the direct current (DC) potential, reduction in EEG amplitude, and often, increases in cerebral blood flow [10–12]. In MA, cortical SD events (CSDs) are thought to occur during the aura phase of migraine and precede the headache phase in a subset of migraineurs [13]. CSD susceptibility (e.g., reduced CSD thresholds) and probabilities of migraine attack are enhanced by increases in cortical excitability [14–16] and result in an increased BOLD signal on fMRI [15, 17–20]. Female reproductive hormones, including estrogen and progesterone, are implicated in migraine development both clinically and pre-clinically [21–23]. Clinically, the onset of migraine attacks coincides with hormonal fluctuations such as those associated with puberty, some oral contraceptives, pregnancy, and menopause [22, 24–26]. Moreover, it is well accepted that migraine without aura can be triggered by steeply declining levels of ovarian hormones, whereas MA is exacerbated by increasing levels of female reproductive hormones [6, 21, 27–30]. Preclinical studies have shown that estrogens, acting through both nuclear α- and β-estrogen receptors as well as GPR30 [31, 32], increase cortical excitability and CSD susceptibility in brain slices [33]. In anesthetized rodent models of CSD, estrogen fluctuations reduced the CSD threshold and increased the frequency and velocity of CSD events [14, 34, 35]. Yet, questions of whether the anesthetic or invasiveness of intracortical recordings altering the CSD events have been raised [36, 37]. Non-invasive epidural EEGs have been used to determine brain activity in rats since 1979 [12, 38–41]. Over time, the implantation technique has been substantially improved upon to yield highly reproducible and stable recordings [12, 41] with successful epidural EEG recordings of CSD events by a number of groups in the context of migraine [42–44]. SD events in humans can be detected with traditional EEG leads on the cortical surface [45] or using scalp AC-EEG [46] intracortical and subdural recordings are only obtained during procedures with patients requiring intracranial pressure relief (i.e., TBI) or tissue removal [47–50].

To date, no studies have investigated the contribution of estrogens to SD induction and headache pain behavior in awake, freely moving, female rats. Here, we addressed this gap in knowledge and study the contribution of 17-β-estradiol to SD and headache pain using our non-anesthetized rodent model [43, 51, 52] including estrogen-receptor dependence and responsivity to sumatriptan, the leading abortive antimigraine. 17-β-estradiol administration induced SDs and behaviors associated with headache pain. Both SDs and pain behavior were inhibited by the non-selective estrogen receptor antagonist/GPR30 agonist ICI 182,780 and sumatriptan, confirming both estrogen receptor dependence and responsivity to the leading abortive antimigraine, respectively.

## RESULTS

### Serum estrogen (E2) levels

Fluctuations in estradiol serum concentrations are associated with CSD and migraine [6, 27]. We injected a supraphysiological dose of 17-β-estradiol (180μg/kg, ip) to maximize the rapid rise and fall of serum estradiol levels in order to capture multiple stages of estrogen activity, including a peak upswing to model pregnancy levels and downfall in levels to mimic sharp fluctuations in estrogen [53], or vehicle (castor oil, ip) 7 days post-OVX or in intact rats. Intact rats served as a control for ovariectomy; high dose estradiol did not effect cytology in any group. Serum samples were drawn before (t=0) and 30 min, 2, 4, 6, 12, and 24 hour after injection to quantify estrogen levels (Table 1). In naturally cycling female rats injected during the diestrus-2 phase of the estrogen cycle, basal serum estrogen levels were 3.53 ± 0.76 pg/mL (n=6). Vehicle injection did not significantly increase serum levels over 24 h as compared to baseline (Table 1, n=3). Administration of 17-β-estradiol (180μg/kg) significantly elevated serum estrogen levels (993.44 ± 498.3 pg/mL, n=3, p<0.01) after 30 min and remained significantly elevated for 4 hours (203.77 ± 50.29 pg/mL, n=3, p<0.0001) before returning to baseline levels after 24 hours (3.29 ± 0.01 pg/mL, n=3) in intact rats. We next determined if 17-β-estradiol administration similarly elevated serum estrogen levels in OVX rats. OVX rats had basal serum estrogen levels of <3.0 pg/mL (n=6) consistent with previous reports and below the limit of detection of our kit (Calbiotech, Spring Valley, CA). Acute administration of 17-β-estradiol elevated serum estrogen concentration that peaked 30 min post-injection (441.35 ± 47.10 pg/mL, n=3, p<0.0005) and remained significantly elevated for 6 hours (164.05 pg/mL ± 48.58, p=0.005). Within 24 h, estrogen levels returned to baseline (4.07 pg/mL ± 0.48, n=3). No significant fluctuation in serum 17-β-estradiol level was observed in vehicle-treated, OVX rats (n=3) at any time over the 24 h post-injection.

**Table 1:** Serum estradiol levels in intact and OVX animals following i.p. administration of 17-β-estradiol (180μg/kg) or vehicle

Surgery	Treatment	BL	30 min	2 h	4 h	6 h	12 h	24 h
**Intact**	**Vehicle**	3.53 ± 0.76	10.24 ± 4.58	8.72 ± 2.64	9.12 ± 2.92	8.26 ± 2.39	8.16 ± 3.75	11.84 ± 5.19
	**17-β**		993.44 ± 498.30	857.50 ± 75.42	203.77 ± 50.29	103.51 ± 36.74	57.63 ± 43.83	3.29 ± 0.01
**OVX**	**Vehicle**	2.57 ± 0.32	< 3.0	< 3.0	< 3.0	< 3.0	< 3.0	< 3.0
	**17-β**		441.35 ± 47.12	204.45 ± 95.55	157.08 ± 77.78	164.05 ± 48.58	8.40 ± 2.5	4.07 ± 0.48

Data are mean ± SEM (pg/mL).

### 17-β-estradiol induces SD events

**Figure 1:**
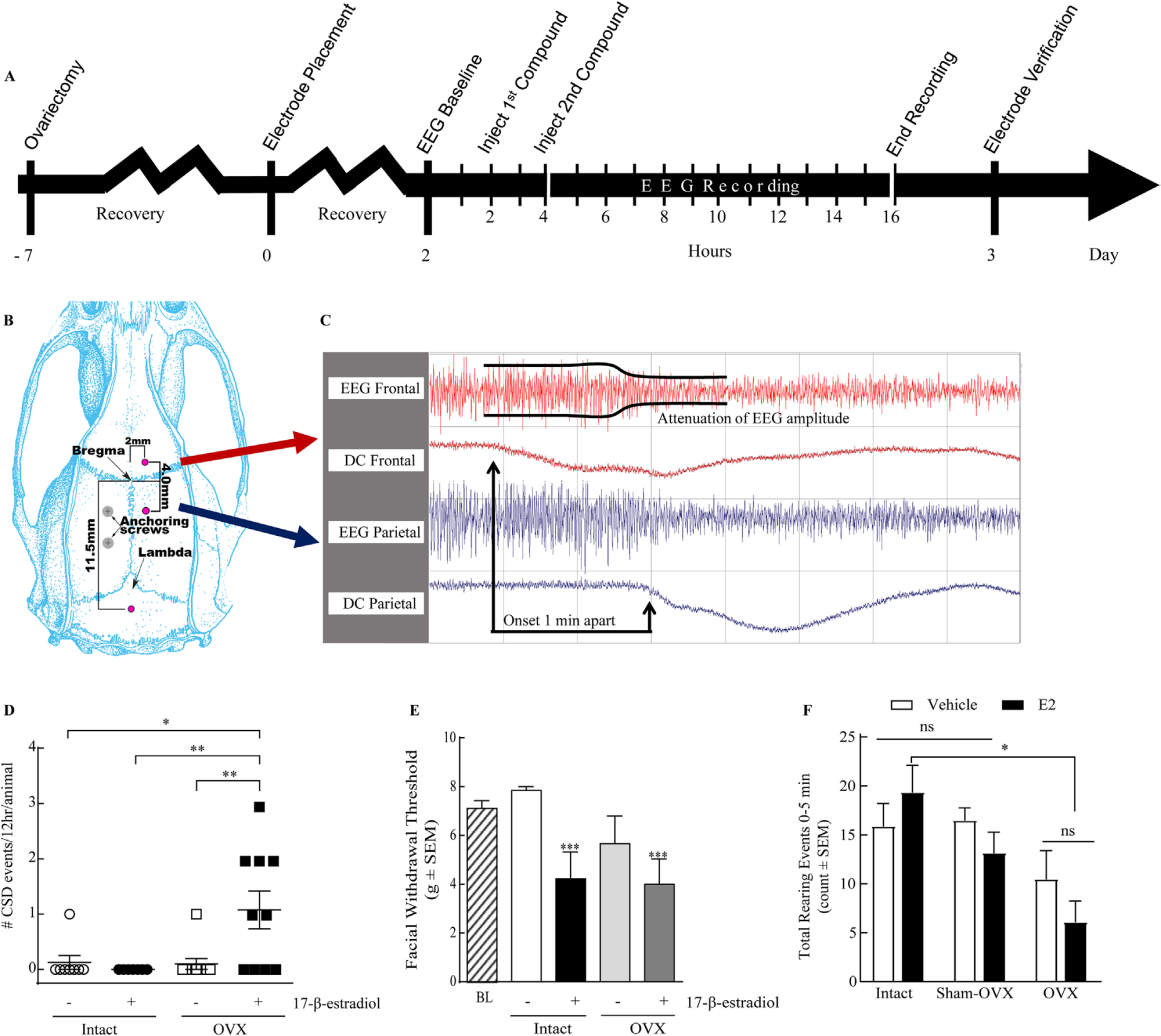
17-β-estradiol administration induces SD events and pain behaviors in ovariectomized rats. **(A)** Schematic timeline of the procedures involved; electrode placement surgery is performed 1 week after ovariectomy and two days before dEEG recording. **(B)** Diagram of the electrode placement surgery. **(C)** A representative dEEG trace displaying a SD event with a propagation speed of 4mm/min. **(D)** 17-β-estradiol induced 1.1 ± 0.35 CSDs per animal (n=10) in ovariectomized rats, while none were elicited in intact rats (n=7). Vehicle had no significant effect on OVX (0.1 ± 0.1 SDs/animal, n=10) or intact (0.13 ± 0.13 CSDs per animal, n=8) rats over 12 hours. **(E)** Facial withdrawal threshold (FWT) is reduced by administration of 17-β-estradiol. Vehicle treated intact rats have a FWT of 7.85 ± 0.16 g (n=8), which is significantly reduced following estradiol administration (4.22 ± 1.11 g, n=8) or OVX and 17-β-estradiol administration 7 days later (3.99 ± 1.04 g, n=9). **(F)** Number of rearing events in 5 min following vehicle or 17-β-estradiol treatment in sham-operated animals was not significantly different from intact animals. 17-β-estradiol treated OVX animals (n=11) have significantly reduced rearing compared to estradiol treated intact animals (n=8). ^*^p<0.05, ^**^p<0.005 as compared to vehicle-treated, intact female rats (Kruskal-Wallis statistic = 26.8; Dunn’s multiple comparisons).

Elevated estradiol levels are associated with an increase in MA frequency but not in migraineurs without aura. Moreover, MA is typically associated with SD events. Therefore, we next asked if 17-β-estradiol induced SD events in awake, freely moving, female rats. Dural electroencephalogram traces were recorded for 12 hours following either 17-β-estradiol (180μg/kg) or vehicle administration, according to the timeline in Figure 1A. Although 1/8 of intact rats treated with vehicle experienced a single SD event, no intact animals treated with 17-β-estradiol had detectable SD events (n=7, Table 2). We also observed a single SD event in 1/10 vehicle-treated animals. In contrast, 6/10 OVX rats (60%) experienced SD events in 12 hours post administration of 17-β-estradiol compared to (Table 2). A total of 11 estrogen evoked SD events observed in 6 OVX rats spread mainly parietal to frontal (8/11); 3/11 traveled frontal to parietal. Overall, 17-β-estradiol SD events propagated at a velocity of 9.26 ± 1.15 mm/min. Negative DC-shifts had amplitudes of 1.30 ± 0.12 mV and durations of 65.01 ± 10.78 s (Table 2). Increases in the number of SDs induced was dependent on the interaction of the gonadal state and administration of exogenous 17-β-estradiol (2-way ANVOA, Interaction: F-value=8.318, p=.007, Figure 1D) suggesting that removal of the ovaries increases susceptibility to 17-β-estradiol-induced SD events.

**Table 2:** CSD characteristics amongst groups

Surgery	Group	% Animals with SD	Speed of propagation (mm/min)	Amplitude (mV)	Duration (s)
**Intact**	**Vehicle**	1/8 (12.5%)	7.70	1.05	43.00
	**17-β**	0/7 (0.0%)	N/A	N/A	N/A
**OVX**	**Vehicle**	1/10 (10.0%)	7.70	2.64	118.00
	**17-β**	6/10 (60%)	9.26 ± 1.15	1.30 ± 0.12	65.01 ± 10.78
	**ICI**	0/6 (16.6%)	N/A	N/A	N/A
	**17-β/ICI**	0/6 (0.0%)	N/A	N/A	N/A
	**Suma**	0/6 (0.0%)	N/A	N/A	N/A
	**17-β/Suma**	0/6 (0.0%)	N/A	N/A	N/A

17-β, 17-β-estradiol; ICI, ICI 732,138; Suma, sumatriptan; OVX, ovariectomy; SD, spreading depression.

### 17-β-estradiol elicits pain behaviors after OVX

Induced SD events are not always associated with pain behaviors such as periorbital allodynia [52], and headache can occur independent from SD. Therefore, we evaluated pain behaviors in intact or OVX rats exposed to 17-β-estradiol or castor oil. Animals were evaluated for periorbital mechanical allodynia with calibrated von Frey filaments; baseline mechanical thresholds were 7.85 ± 0.15 g. Castor oil (vehicle) had no effect on facial sensitivity in naturally cycling (n=8) as compared to baseline values but administration of 17-β-estradiol significantly reduced mechanical threshold at 2 h in 5/8 intact rats (4.22 ± 1.11 g, p<0.05). Although OVX alone reduced facial withdrawal thresholds in 3/8 rats as compared to baseline values, overall hypersensitivity was not significantly different. 17-beta estradiol administration further reduced facial withdrawal thresholds in 6/9 OVX rats (OVX: 3.99 ± 1.03 g, p<0.005; Figure 1E). These data suggest that while loss of ovaries increases the percent of animals experiencing periorbital allodynia, administration of 17-β-estradiol induced periorbital allodynia regardless of gonadal state.

In addition to periorbital allodynia, suppression of exploratory behaviors was recently linked to preclinical migraine [54–56]. We determined if total times of exploration, ambulation, and fine movement, as well as rearing events, were decreased in rats 2 hours after exposure to high dose 17-β-estradiol or vehicle. Exploratory behaviors such as transitional exploratory events (i.e., chamber to chamber), ambulatory events, and fine movements were not significantly different between 17-β-estradiol and vehicle controls suggesting that these behaviors were not suppressed (p = 0.05, 0.12, and 0.25, respectively) 2h after administration of 17-β-estradiol. The total number of rearing events was assessed over 30 minutes, 2 h after vehicle or 17-β-estradiol administration. Rearing behavior was not significantly different between intact vehicle- and 17-β-estradiol- treated animals (52.73 ± 10.74, n=11 and 44.30 ± 6.21, n=10 respectively, p=0.85 Bonferroni). The total number of rearing events over a 30 min time course was significantly reduced in OVX vehicle treated rats (19.38 ± 5.23, n=8, p<0.05) and for OVX animals treated with 17-β-estradiol (9.857 ± 1.98, n=7, p<0.005 Bonferroni) compared to intact vehicle treated animals.

We next assessed if rearing reductions in OVX animals were due to exacerbation of post-surgical pain or a result of hormone fluctuations. A separate set of animals was generated in which sham-OVX surgeries were performed with rearing assessed before surgery and in the presence and absence of 17-β-estradiol (180 μg/kg). The total number of rearing events observed after sham-OVX + vehicle was not significantly different from intact, vehicle treated rats (Figure 1E, 64.5± 7.7, n=6 vs. 52.7 ± 10.7, p>0.99, Bonferroni). Likewise, administration of 17-β-estradiol to sham-OVX rats did not significantly reduce rearing over the 30 min observation period (33.5 ± 5.6, p>0.99, Bonferroni). Thus, suppressed rearing observed in OVX + 17-β-estradiol animals was not likely due to exacerbation of post-surgical pain.

To determine if reduced rearing correlated to exploring new environment, we assessed events in 5 min bins. Most rearing events occurred in the first 5 minutes (Figure 1F), consistent with exploring a new environment and subsided to very few (i.e., 0-2 events) over the 30 min regardless of treatment. Consistent with the total rearing data, both vehicle (8.25 ± 1.79, n=8, p<0.05; Figure 1C) and 17-β-estradiol (3.00 ± 0.65, n=7, p<0.005) treated OVX animals reared less over the first 10 min as compared to intact, vehicle-treated rats. No differences were seen between sham-OVX rats and intact rats at any time point. Suppression of rearing was dependent on gonadal state only. These data, together with periorbital allodynia, indicate that 17-β-estradiol induced distinct manifestations of pain behaviors in both intact and OVX rats.

### 17-β-estradiol evoked CSD events and suppressed rearing requires estrogen receptors

**Figure 2:**
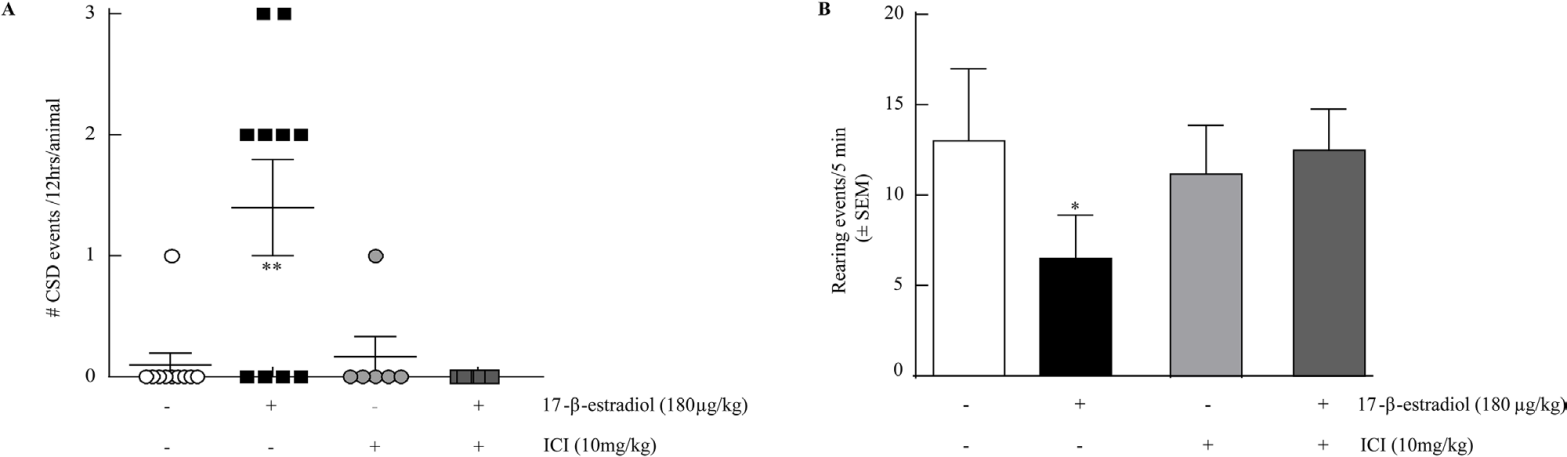
ICI 182,780 prevents estrogen induced SD and estrogen induced reduction in rearing. **(A)** 17-β-estradiol 180μg/kg induces SD events in OVX rats over 12 hours (1.4 ± 0.4 SDs, n=10) which is inhibited by ICI 182,780 10mg/kg, i.p. (0 ± 0 SDs, n=10). **(B)** 17-β-estradiol reduction of rearing behavior over 5 minutes (n=6) is inhibited by pretreatment with ICI 182,780 10mg/kg, i.p. (n=6). ^*^p<0.05; ^**^p<0.005 (One-way ANOVA).

Focusing on the OVX rats, which had a significantly higher number of SDs and prominent pain behavior, we asked if estrogen receptor activation was required for 17-β-estradiol-induced SDs and suppression of rearing behaviors. We administered the estrogen antagonist ICI 182,780 (10 mg/kg, i.p.) 2 hr before 17-β-estradiol or vehicle. 17-β-estradiol evoked SD events were blocked in the presence of ICI 182,780 (Table 1, Figure 2A). Importantly, ICI 182,720 did not elicit SD events when administered alone. Similarly, pretreatment with ICI 182,780 significantly prevented 17-β-estradiol-induced suppression of rearing events in rats (12.5 ± 2.26, n=6, p<0.05; Figure 2B) within the 0-5 min period. Thus, 17-β-estradiol induced SD events and pain behaviors required activation of estrogen receptors in OVX rats.

### Sumatriptan prevents 17-β-estradiol evoked CSD events and suppressed rearing

**Figure 3:**
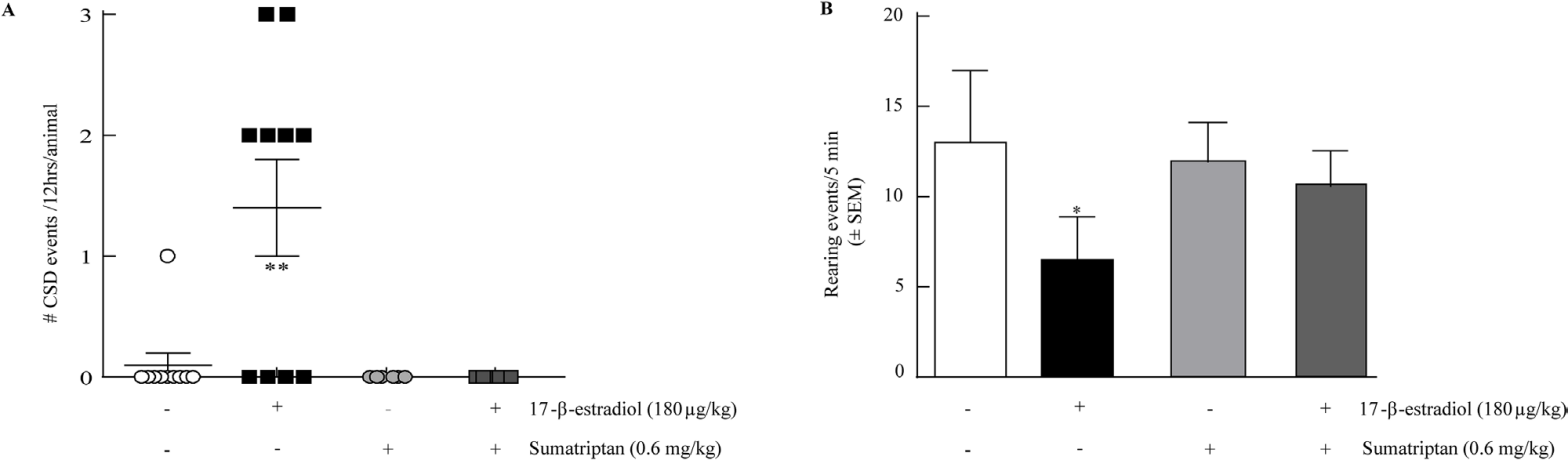
Sumatriptan prevents estrogen induced SD and estrogen induced reduction in rearing. **(A)** Sumatriptan 0.6mg/kg, i.p. (0 ± 0 SDs, n=6) inhibited 17-β-estradiol induced SD events in 12 hours with 2 h pretreatment. **(B)** 17-β-estradiol suppression of rearing behavior over 5 minutes (n=6) was inhibited by 2h pretreatment with sumatriptan 0.6mg/kg, i.p. (n=6). ^*^p<0.05, ^**^p<0.005 (One-way ANOVA).

We assessed the ability of the abortive anti-migraine therapeutic sumatriptan (0.6mg/kg, i.p.) to prevent 17-β-estradiol SD events or rearing behaviors. Pre-treatment with sumatriptan 2 hours before 17-β-estradiol prevented the induction of SD events (n=6; Table 1, Figure 3A) and did not induce SDs on its own. Since the majority of rearing events occurred within the 0-5 min bin of the 30 min total observation period, we evaluated the effects of sumatriptan intervention within this time-period. Sumatriptan pretreatment increased the number of rears in 17-β-estradiol treated OVX animals (10.6 ± 1.95, n=10) back to that of OVX-vehicle treated rats (12.27 ± 3.14, n=6, p=0.21; Figure 3B).

## DISCUSSION

Migraine with aura affects nearly 8% of migraineurs, the majority of whom are female [57–59]. In addition to the aura and headache, the patients suffer from a greatly diminished quality of life as evidenced by a greater absenteeism from work/school, less productivity, and less overall energy [60]. Increases in estrogen levels are implicated in the development of MA, but preclinical studies of this phenomenon in female subjects are few [6]. Here, we examined the role of 17-β-estradiol in the induction of SD events and headache-like pain, two characterizing features of MA, in non-anesthetized female rats. We show that exogenous application of 17-β-estradiol elicits SD events and corresponding pain behaviors in a gonadal state (intact versus OVX) dependent manner. These events were prevented with both estrogen receptor antagonist and administration of the anti-migraine therapeutic sumatriptan.

Women are 3 times more likely to suffer from migraine headaches than males with reports of higher pain levels and incidence of aura [5, 61–64]. Plasma estradiol and progesterone levels in non-migrainous women not taking hormonal birth control are lower throughout the menstrual cycle compared to migraineurs [65], implicating these hormones in migraine pathology. Interestingly, female migraineurs without aura taking combined oral contraceptives (i.e., those containing estrogen) reportedly have increased migraine attacks in between courses- when serum estrogen levels drop or are at the lowest point [66]; serum estradiol levels peak following administration of oral contraception between 100-120 pg/ml [67–69]. In contrast, in migraineurs with aura, contraceptives with estrogen are associated with increased frequency and severity of migraine attack as well as increased risk of ischemic stroke [70] and are thus contraindicated; conversely, progesterone-only pills are not [71]. These clinical findings highlight the idea that fluctuations in ovarian hormones (e.g., estrogen) can differentially affect migraineur sub-populations [62].

In the present study, we found that a single supraphysiological dose of 17-β-estradiol (180 μg/kg) increased serum concentrations of 17-β-estradiol in both intact and OVX rats for up to 6 hrs. Observed serum 17-β-estradiol levels were supraphysiological in animals mimicking reproductive (intact) and non-reproductive states (OVX) at values in line with patients with gonadotropinomas and other anterior pituitary tumors [72, 73]. This use of a bolus, supraphysiological dose allowed for assessment of SD events and pain behaviors across a sharp increase and decrease in estrogen levels During migraine *without* aura, these can be times of headache relief. However, elevated levels of estradiol in women with MA, are associated with increases in migraine frequency and severity [74] and induction of CSD events [6, 14, 15, 19, 75]. In our current investigation, SD events occurred 30 min after 17-β-estradiol administration in OVX rats suggesting that changing estrogen levels were required to induce SD events; this was dependent on gonadal state and administration of 17-β-estradiol. Interestingly, SDs persisted after estradiol levels had returned to baseline values in some rats (up to 12 hours) indicating an increased susceptibility to neuronal dysregulation after 17-β-estradiol. These observations align with work done by Sachs and colleagues showing acute estrogen alone was able to induce CSDs in rat neocortical slices [33], as well as studies showing that chronic estrogen can reduce the threshold for initiation of KCl-induced CSDs in anesthetized, OVX rats [34]. Likewise, our findings align with Chauvel et al., showing that OVX plays a role in SD susceptibility and frequency in non-anesthetized, female rats after 17-β-estradiol.

CSD events are linked to the aura that precedes migraine headache in 30% of migraineurs and are separate phenomena from headache-like pain behaviors [43]. In assessing headache pain behaviors, this investigation shows that 17-β-estradiol induces periorbital allodynia in both naturally cycling and OVX rats to similar degrees. However, 17-β-estradiol only suppressed spontaneous rearing behavior in OVX rats. Although OVX-vehicle treated rats reared less than intact rats at baseline, this was not an exacerbation of post-surgical pain [76, 77]. Our observations align with a recent study by An et al., showing that estrogen administered to OVX rats enhanced incision evoked tactile sensitivity [78] and suggests that estrogen fluctuation coupled to state of intactness may contribute to pain sensitivity. Given that periorbital allodynia (a reflex) and rearing (a spontaneous behavior that is suppressed during pain) represent two independent perceptions [79], it is plausible that the underlying mechanisms driving the two behaviors in the presence of E2 differ and account for the different presentations observed in intact and OVX rats. While we observed periorbital allodynia for all rats receiving 17-beta-estradiol, not all migraineurs experience this symptom [80]. Interestingly, presence of allodynia in patients corresponded with higher disability (suppression of normal behaviors); it is more prevalent in migraineurs with aura. These clinical observations may explain the dissociation of pain behaviors we observed (i.e., rearing behaviors only in OVX animals) and indicate a heterogeneous presentation of estradiol-induced head pain in rats.

Estradiol acts at nuclear α- and β-estrogen receptors as well as the membrane receptor GPR30 [31, 32]. These estrogen receptors are located throughout the craniofacial pain axis and the central nervous system [81–84]. Fluctuations in estrogen levels or total loss of endogenous estrogens can lead to changes in receptor expression in migraine relevant regions [82, 85–87]. We show that the non-selective estrogen receptor antagonist ICI 182,780 effectively prevented the induction of SD events and suppressed rearing behavior by 17-β-estradiol suggesting that estrogen receptors are required for these phenomena; identification of receptor sub-types responsible will require additional studies beyond the scope of this paper.

Triptans are first-line, abortive therapies for episodic migraine associated with high therapeutic gains and favorable side-effect profiles [88–91]. In the current study, we showed that pretreatment with sumatriptan prevented 17-β-estradiol induced SD events. In contrast to our observation, acute sumatriptan did not reduce cGMP- or KCl-induced CSD events in previous studies [92, 93]. Interestingly, sumatriptan effectively slowed propagation and reduced the amplitude/duration of retinal SD events *in vitro* [94, 95] suggesting that sumatriptan is capable of suppressing SD events. Differences in observations as reported within the migraine literature may reflect variability in subject sex, gonadal state (intact versus OVX), alertness (i.e., anesthetized/awake), model of SD induction (multiple/single events), and/or CNS penetration of sumatriptan under experimental conditions [89, 93, 96–99]. In addition to preventing SD induction, sumatriptan restored natural rearing behaviors to OVX, 17-β-estradiol treated rats suggesting that this behavior was sensitive to migraine therapeutics and relevant toclinical manifestations of pain [60, 91–93, 100, 101]. These data are consistent with reports that triptans need to be on-board during prodrome for effect in migraineurs with aura, and that triptans are ineffective when taken during the headache phase [88, 91, 102–106].

### Limitations of this study

While we provide a novel model of hormone-mediated SD sensitivity, a few limitations to this study exist. First, although EEG recordings have been shown in animals and humans to represent a SD event, we do not have direct evidence using intracortical recordings that observed SD are cortical. In two animals (intact + vehicle and OVX+ vehicle), a single SD event was observed. While it is not possible to rule out induction of events in these animals due to endogenous factors such as the innate stress response or focal ischemia [107–109], no events occurred during the 2 h baseline recording nor at injection times suggesting that the stress associated with experimental execution was not responsible. Given that focal ischemia tends to produced negative shifts in DC potential of approximately 20mV in cortical recordings or 1-5mV shifts coupled with reduction in epidural EEG amplitudes during recordings [12], and our observed negative shifts were 1.05 and 2.64 mV, respectively, we cannot rule out a focal ischemic event triggering the observed SD events in these 2 rats. However, “energy–compromised” tissue (e.g., ischemic) induced events are typically evidenced by non-spreading depression or by peri-infarct depression where a negative shift in DC potential is not coupled to a reduction in ECoG amplitude [12]. Second, intact rats were assessed only in diestrus when estrogen levels are at their lowest [6, 110]; only OVX rats exhibit estradiol-induced SDs lending credence to use of intact rats during this phase. This provides a proof of concept that female sex hormones can induce highly reproducible SD. Third, we used a high dose of estradiol to induce SD events; this does not preclude the efficacy of lower doses in this model. Fourth, the acute administration and rapid onset of 17-β-estradiol in our model does not replicate most migraineurs but does allow for the induction and analysis of SD across estrogen peaks and falls.

## MATERIALS AND METHODS

### Animals

Female, Sprague Dawley rats (275-300g) purchased from Harlan (Indianapolis, IN) were housed in a climate controlled room on a regular 12 hour light/dark cycle with lights on at 7:00 am with food and water *ad libitum*. All procedures were performed during the 12-hour light cycle and according to the policies and recommendations of the International Association for the Study of Pain, the NIH guidelines for laboratory animals, and were approved by the IACUC of the University of Arizona.

### Drugs

Ketamine/xylazine was purchased from Western Medical Supply (Arcadia, CA). 17-β-estradiol, ICI 182,780 and sumatriptan were purchased from Tocris (Ellisville, MO). 17-β-estradiol and ICI 182,780 were dissolved in castor oil to appropriate concentrations the day of experiments. Sumatriptan was dissolved in saline. Castor oil and sterile saline (0.9%) served as appropriate controls. All drugs were administered via intraperitoneal (i.p.) injections.

### Removal of ovaries

Rats were anaesthetized with ketamine:xylazine (dose: 80:12 mg/kg, 1ml/kg) and ovariectomized [54]. The ovaries were removed via a bilateral side approach, whereby a 3-5 mm incision is made through the skin, fascia and muscle. Ovarian arteries were ligated and the ovary excised. Prophylactic gentamicin 8mg/mL, 1mL/kg was delivered i.p. following surgery. The rats recovered for 7 days before implantation of recording electrodes and/or any experiments (Figure 1A).

### Vaginal smears

Estrous cycles of intact female rats were monitored by daily vaginal smears. The vaginal smears were interpreted as described by Goldman et al [110]. Briefly, vaginal openings were flushed with 200 μL of sterile saline. Fresh samples were evaluated for cytology at the same time daily for 8 days using a Zeiss Axioskop 40 (10x/0.3 numerical aperture EC Plan-Neofluar objective). CSD events, serum concentrations, and pain behaviors were assessed in the morning on diestrus day 2 by cytology.

### Serum estrogen concentration

Serum concentrations of E2 before and at 30 min, 2, 4, 6, 12 and 24 hours post injection of either a dose of 17-β-estradiol (180 μg/kg, i.p., n=3/time-point) [55, 113] or vehicle (castor oil, 1ml/kg) were determined. Briefly, blood samples were drawn via intracardiac puncture under isoflurane anesthesia (5% induction, 2.5% maintenance in air- 2L/min); animals were then decapitated. Samples were allowed to clot for 30 min at RT then spun in microcentrifuge tubes at 10,000 RPM for 30 min at 4°C to separate serum from erythrocytes. Serum was collected, flash frozen in liquid nitrogen, and kept frozen at -80°C until day of concentration determination. An enzyme linked immunosorbent assay (ELISA) kit for rat estrogen (E2) was purchased (Calbiotech, Spring Valley, CA) and performed according to manufacturer’s instructions.

### Implantation of recording electrodes

Silver chloride (AgCl) electrodes were prepared by flaming 0.25mm Ag wire (A-M Systems, Inc., Everett, WA) into spherical tips (1mm diameter) and subsequently coating the tips with chloride as previously reported [43]. Rats were anaesthetized with ketamine/xylazine, as above, 7 days post-ovariectomy. The rats were fixed to a stereotaxic frame (Stoelting) and three burr holes were drilled through the skull using a manual drill to allow placement of the AgCl recording electrodes. The frontal and parietal lead electrodes were placed in the right hemisphere 2 mm lateral and 1.5 mm anterior to bregma and 2 mm lateral and 2.5 mm posterior to bregma, respectively; the reference electrode was placed 7.5mm posterior to bregma (Figure 1B). Two screws (#MPX-080-3F-1M, Small Parts Inc., Miami Lakes, FL) were fastened into the left hemisphere of the skull separated by 2mm without going through the skull. The front screw served as a mounting support to assist in anchoring the multi-pin connector. The back screw served as a ground electrode, which was made by soldering silver wire onto the head of the screw. The four electrodes (three silver chloride electrodes and one ground electrode) were soldered into the bottom of a multi-pin connector (Continental Connector, Hatfield, PA) and the apparatus was fixed into place using dental cement.

### Electrophysiological recordings

Forty-eight hours after dural electrode implantation, rats were placed in a recording chamber (40 cm L x 49 cm W x 37 cm H) and the multi-pin connector attached to an electro-cannular swivel (#CAY-675-6 commutator, Airflyte, Bayonne, NJ) mounted in the ceiling of the chamber. The swivel allowed rats to move freely about the chamber during the recording period. Animals habituated to the chamber for two hours prior to any pharmacological intervention to permit electrical recordings to stabilize. Only those rats with stable electrical recordings were included in experimental groups. Signals led to separate DC and AC amplifiers (Grass Model 15 amplifier system, 15A12 DC and 15A54 AC amplifiers, West Warick, RI) through insulated cables and collected with dEEG recording analysis software Gamma v.4.9 (Astro-Med, Inc. West Warick, RI). Following 17-β-estradiol or vehicle injection, the rats were observed for 12 hours without interruption. Subsequent to dEEG recordings, animals were sacrificed and cemented electrodes were carefully removed to ensure there was no damage to the dura. Data from rats with damaged dura were excluded from analysis.

### Electrophysiological recording analysis

Following electrophysiological recording, the data were reviewed with Gamma Reviewer (Astro-Med, Inc. West Warick, RI) and analyzed for the SDs offline. SD events were determined by the DC shifts that were calculated from both the frontal and parietal electrodes that recorded the shifts. Cortical activity was defined as depressed during these events by measuring the significant reductions in both the power and amplitude of the dEEG tracings (i.e., recorded electrical activity was only considered an SD event when in both frontal and parietal electrodes: 1) the AC current was reduced by half; 2) the DC current exhibited a downward shift by a minimum of 1mV; and 3) for a minimal duration of 30 s [12, 41, 42, 44, 50]; Figure 1C). The velocity of SD propagation was determined by analyzing the difference in time of onset of DC current depression between the frontal and parietal electrodes and dividing into 4mm (the distance between the two electrodes).

$$\text{SD velocity}=\frac{4\mathrm{mm}}{\left({\text{depressiononset}}_{\text{diff}}\right)}\times \frac{60\;\sec}{1\min}$$

### Activity testing

Activity testing was conducted using behavioral techniques similar to those previously described [43, 51, 56, 114, 115]. Rats were acclimated to the testing room, but not to the activity recording chambers for 1 hour prior to injection. Rats were then administered an estrogen antagonist (ICI 182,780), sumatriptan, or vehicle 2 hours prior to 17-β-estradiol or vehicle injection. Two hours after the second injection, animals were placed in individual recording chambers (41 cm long X 41 cm wide X 39 cm high, Tru Scan Photobeam system: Coulbourn Instruments or 27.5 in long X 8.75 in wide X 13.125 in high, Place Preference system: San Diego Instruments). Each Tru Scan chamber has 2 photobeam sensor rings that measure all movements in 3 dimensions. One ring is located near the floor of the chamber measuring all horizontal movements including time, distance, and velocity of movements. The second ring is at the same fixed height across all chambers and records the vertical movements (i.e. rearing behavior) including the number of plane breaks and time the rat is vertical. Locomotor/exploratory activity was recorded and analyzed using Tru Scan software v.3.11 (Coulbourn Instruments). The Place Preference system records movement with a 4 X 16 photobeam array providing x,y movement, captured into PAS Software (San Diego Instruments) which displays the information as exploration, ambulation, and fine movements. Vertical rears were counted each time a rat stood on both hind paws without grooming.

### Periorbital mechanical allodynia

Rats were acclimated to testing box 1 hour prior to evaluation of periorbital mechanical allodynia with von Frey filaments as previously described [43]. Behavioral responses were determined by applying calibrated von Frey filaments perpendicularly to the midline of the forehead at the level of the eyes with sufficient force to cause the filament to slightly bend while held for 5 seconds. A response was indicated by a sharp withdrawal of the head. Mechanical thresholds were evaluated at baseline, 6 days post OVX before drug administration, and 2 hours post 17-β-estradiol (180 μg/kg) or vehicle (castor oil, 1ml/kg) administration by an observer blinded to drug administration.

### Statistical analysis

Prism software was employed to perform statistical analysis. All data are expressed as mean ± standard error of the mean (SEM). SD data was statistically compared using a one-way ANOVA and Student’s-Neuman-Keul’s post-hoc test. Statistical significance of periorbital allodynia was determined by repeated measure two-way ANOVA to analyze differences among treatment groups with a Bonferroni test applied post-hoc. In rearing experiments, data were expressed as mean ± SEM. A one-way ANOVA, Bonferroni post-hoc was used or an unpaired t-test with Welch’s (2 group) correction to determine statistical significance. Data were accepted as statistically significant when p ≤ 0.05.

## CONCLUSION

While estrogen withdrawal is associated with induction of migraine without aura in humans [67], elevated estradiol likely contributes to MA. Here, utilizing awake adult-female rats measuring both pain behaviors and cortical spreading depression as a marker for MA, we link 17-β-estradiol levels [111, 112] to induction of CSD events and generation of pain behaviors in OVX rats. Our studies validate 17-β-estradiol as an exogenous stimulus that recapitulates several aspects of clinical migraine with aura including induction of pain behaviors and therapeutic responsiveness suggesting that both the direction, rate of change, and basal set point of estrogen levels may influence migraine pathogenesis [14, 43].

## Abbreviations

CSD: Cortical spreading depression
DC: direct current
EEG: electroencephalogram
E2: estradiol
dEEG: dural electroencephalogram
HRT: hormone replacement therapy
MA: migraine with aura
OVX: ovariectomy
AgCl: silver chloride
SD: spreading depression
